# Exosomal lncRNAs: the newest promising liquid biopsy

**DOI:** 10.20517/cdr.2019.69

**Published:** 2019-12-19

**Authors:** Marta Tellez-Gabriel, Dominique Heymann

**Affiliations:** ^1^RNA and Molecular Pathology (RAMP) Research Group, Department of Medical Biology, The Artic University of Norway (Tromso), Tromso 9019, Norway.; ^2^INSERM, Institut de Cancérologie de l’Ouest, LabCT, U1232, CRCINA, Université de Nantes, Université d’Angers, Saint Herblain 44805, France.

**Keywords:** Liquid biopsy, long non-coding RNAs, exosomes

## Abstract

LncRNAs are defined as RNA transcripts greater than 200 nucleotides in length that have no or limited protein-coding potential. Basal expression of lncRNAs appeared important for various homeostatic processes, like gene imprinting cell differentiation and organogenesis. Moreover, it has been demonstrated that lncRNAs play an important role in tumorigenesis and metastasis. Some lncRNAs were stably detected in exosomes, which are widely found in body fluids. Several studies validated the use of exosomal lncRNAs as minimally invasive diagnostic and prognostic markers in several types of cancers. In addition, exosomal lncRNAs have been associated with drug resistance of tumor cells, suggesting a clinical application in cancer-targeted therapy. Despite the recent increase of studies on exosomal lncRNAs, their clinical significance in cancer diagnosis, prognosis and treatment needs to be fully explored. The methodologies for their detection with high purity and accuracy must be also improved in order to implement their use in clinical routine. This review aims to summarize the main recent technologies available for the isolation of exosomal lncRNAs, their status as a liquid biopsy as well as their future perspectives.

## Introduction

LncRNAs are classically defined as RNA transcripts greater than 200 nucleotides in length that have no or limited protein-coding potential^[1]^. The location of a lncRNA is often considered relatively to its neighboring protein-coding gene(s). LncRNA genes overlapping with protein-coding genes are antisense lncRNAs; intronic lncRNAs are located in the introns of protein-coding genes; lncRNA genes in the intergenic genomic loci are termed lincRNA. In addition, some genes encode both protein-coding and lncRNA genes^[2]^. In recent years, lncRNA were identified to regulate gene expression through various mechanisms^[3]^. Basal expression of lncRNAs in many tissues regulates various homeostatic processes, including gene imprinting, cell differentiation and organogenesis^[4,5]^. There is also a strong association between deregulated lncRNA expression and disease evolution. Indeed lncRNAs play an important role in tumorigenesis and metastasis^[6,7]^. For instance, in liver, gastric and breast cancers, GAS5 and HULC lncRNAs are involved in the control of cell invasion by regulating miRNAs and the interactions between tumor microenvironment, and cancer cells^[8-10]^. LncRNAs are then expressed abundantly by cancer cells and show greater tissue-specificity compared to protein-coding mRNAs. Thus, expression levels of lncRNAs may represent a powerful biomarker^[11]^.

LncRNAs are localized in either the nucleus or cytoplasm, and can interact with DNA, RNA or proteins^[12]^. To exert their function, lncRNAs interact in both sequence-specific and conformational engagements acting as scaffolds, decoys (molecular sink/miRNA sponge) and enhancer RNAs^[11]^. Interestingly, lncRNAs can also be packaged into exosomes and act as messengers in cell-to-cell communications^[13]^. Notably, some lncRNAs are enriched in exosomes, while others are barely present, indicating that lncRNAs are selectively sorted into exosomes. Specific proteins might act as fundamental lncRNAs carriers to control lncRNA release into exosomes. Unfortunately, the exact mechanisms associated with this regulation are not yet fully elucidated. Recently, it has been demonstrated that exosome-derived lncRNAs regulate tumor cell apoptosis, proliferation and migration and induce angiogenesis^[14,15]^.

The influence of exosomal lncRNAs in the tumor microenvironment has been recently investigated. Cancer and stromal cells use exosomes to modify surrounding cells within tumor microenvironment by transferring ncRNAs and proteins, which activate signaling pathways via receptor-ligand interactions and contribute to tumor progression and metastasis^[16]^. In addition, exosomes modulate the escape of cancer cells from immune cells by releasing immunoregulatory molecules. For example, Qu *et al.*^[17]^ found a high expression of lncARSR in sunitinib-resistant RCC cells, which were able to disseminate survival skills to other recipient cells via exosomes containing lncARSR^[17]^. Some studies showed that lncRNAs secreted by tumor-derived exosomes could stimulate the proangiogenic potential of circulating angiogenic cells by increasing expression of both membrane molecules and soluble factors^[18]^. In this regard, Ma *et al.*^[19]^ demonstrated a high expression of HOTAIR lncRNA in glioma cells. Interestingly, HOTAIR is packed into exosomes secreted by glioma cells and transmitted to endothelial cells where it stimulates angiogenesis by increasing the expression of the proangiogenic factor VEGFA^[19]^. Interactions between metastatic cells and their microenvironment via lncRNA-containing exosomes were also demonstrated. High expression of exosomal HOTAIR was correlated with tumor migration and metastasis in several studies^[20-22]^. In addition, lncRNAs secreted by exosomes can regulate tumor stem cell characteristics. For instance, Xu *et al*.^[23]^ showed that the GAS5 lncRNA controls human embryonic stem cell self-renewal^[23]^.

The relationship between exosomal lncRNA and cancer disease progression as well as exosome isolation from liquid biopsies make exosome-derived lncRNAs excellent candidates as potential diagnostic and prognostic biomarkers of various cancers^[24,25]^. The present review aims to summarize the main recent technologies available for the isolation and detection of exosomal lncRNAs and their status as liquid biopsies. Moreover, it will focus on future perspectives.

## RNA based liquid biopsies: exosomal lncRNAs

In the field of biomarkers, RNA exhibits some advantages over DNA. First, the expression pattern of several RNA molecules, in particular lncRNAs, is highly tissue- or disease-state-specific. In addition, RNA expression is dynamic and fluctuates according to the internal needs of cells^[26]^. Finally, RNA analysis allows the detection of several molecules such as non-coding RNAs, fusion transcripts, splice variants and RNA editing events.

Liquid biopsy compared to traditional solid biopsy has been widely used as a noninvasive diagnosis and molecular phenotyping technology to detect early cancer states. Liquid biopsy has been also proposed to determine tumor dynamics and can provide diagnostic and prognostic information prior to treatment, during treatment and during progression^[27]^. The ideal cancer biomarker must be able to identify the cancer subtype, the stage of the disease, the most efficient therapy and to predict/detect tumor recurrences. Moreover, it should be detected by a noninvasive method applicable into clinical routine. Thanks to their specificity, lncRNAs detectable in body fluids have the potential to meet these expectations, but some essential obstacles still need to be addressed.

Exosomes are extracellular vesicles that are 40-100 nm in diameter with a two-layer lipid structure. The formation process of exosomes mainly relies on the endocytosis of cell membrane to form endosomes. Exosomes contain large amounts of nucleic acids (DNA and ncRNAs, including mRNA, miRNA, and lncRNA), proteins and lipids. They act on the recipient cells by carrying these substances^[28]^. These extracellular vesicles are widely found in body fluids, including blood, tears, urine, saliva, milk, ascites, *etc*.^[29]^. Cancer cells secrete more exosomes than normal cells, and the biological information of cancer can be directly obtained by analyzing cancer-derived exosomes^[30]^. Exosomes are involved in critical processes of cancer development, including tumor growth, metastasis, drug resistance and tumor microenvironment^[31,32]^. Exosomal lncRNAs are differently expressed in many cancers suggesting that they could reflect the physiological and pathological status of their parental cells^[24,33]^. Moreover several studies reported great differences in the expression profiles of exosomal lncRNAs between healthy people and cancer patients^[34,35]^, as well as among cancer patients at different stages of the disease^[36]^. This tumor-association specificity has attracted attention in precision oncology as promising biomarkers for cancer diagnosis as well as ideal prognostic biomarkers for cancer management. Interestingly, Dong *et al.*[37] examined the RNA content in exosomes, apoptotic bodies, microvesicles in blood, and found that lncRNAs in blood are mainly distributed in exosomes, suggesting that lncRNAs could be secreted into the blood by the form of extracellular vesicles^[37]^. The exosomal lncRNAs appear stable in other body fluids, such as urine, alveolar lavage fluid, saliva, *etc*.^[38]^.

Compared to circulating tumor cells and free nucleic acids, exosomes are more stable and they are found in a higher concentration^[39,40]^. Recent findings by Matsumoto *et al*.^[41]^ indicate that integrin CD47 expression on the exosomal surface would protect them from phagocytosis by monocytes and macrophages^[41]^. Thanks to their small size, exosomes can easily penetrate into various body fluids and reflect the real-time status of a live body. Finally some specific diagnostic biomarkers can be only detected in circulating exosomes thanks to their enrichment inside these vesicles, since normally they would not be detected in other liquid biopsies, such as free nucleic acids, due to their low expression values^[42]^. Hence, circulating exosomal lncRNAs are considered as a promising liquid biopsy for cancer diagnosis, prognosis monitoring, and cancer treatment. However, compared to other liquid biopsies such as CTCs and ctDNA, the research and application of exosomal lncRNAs are still in their infancy.

## Technologies for the isolation and detection of exosomal long noncoding RNA

Isolation of exosomes is the first step in order to purify them and to obtain enough quantities to perform a proper analysis of their content (e.g., proteins, RNA or DNA). Various methods have been developed for isolating exosomes from biological fluids. Here we will review the main methodologies currently available as well as their major advantages and disadvantages [Table 1].

**Table 1:** Methods of exosome isolation

Method	Principle	Advantages	Disavantages
Differential centrifugation	- Alternative low/high-speed centrifugation steps - Size based separation	- Simple operation, no contamination by separation reagents, high yield, cheap	- Viscosity affects drastically the efficiency, exosome disruption
Density gradient centrifugation	- Combine ultracentrifugation and sucrose density gradient - Density based separation	- High purity	- Difficult to operate, very sensitive to centrifugation time
Size-exclusion chromatography	- Polymer column packed with porous beads - Size based separation	- High purity - Purified exosome structure well preserved - Compatible with different elution solutions	- Time consuming - Low concentrated exosomes obtained
Ultrafiltration	- Membranes with various pore size - Size based separation	- Simple to operate - Low volume of initial sample - High concentrated exosomes	- Exosomes may stick to the membranes - Exosomes structure may be affected - Low purity
Polymer-based precipitation	- Polymer precipitation solution followed by low-centrifugation - Size and density based separation	- Mild effect on exosomes - Different volumes and biological fluids can be used - Fast, high yield and cheap	- Exosome aggregates, polymers may interfere with downstream analysis - Low purity
Immunoafinity	- Antibody coated beads and magnets - Specific protein expression based separation	- Very high purity and selectivity	- Expensive - Small sample volumes can be processed - Low yield and nonspecific binding - Exosomes can remain stuck to the beads
Microfluidic based on-a-chip systems	- Specific surface markers, size and density or other physical properties	- Integration of multiple processes in one device - High accuracy - Precise control - Minimal sample size	- High physical knowledge required to design and operate these devices

The most commonly used method for exosome extraction is differential centrifugation. By using low-speed and high-speed centrifugation alternately, vesicle particles of similar size can be separated from cells and cell debris. Viscosity of the biofluids is the most important parameter to determine time and speed of the different centrifugation steps and collect isolated exosomes with high purity^[43]^.The main advantages of this method are: (1) simple operation; (2) no contamination by separation reagents; (3) large amounts of exosomes obtained; and (4) its low cost. However, the efficiency of this method is reduced when high viscosity fluids such as plasma or serum are used and shearing forces may cause disruption.

Density gradient centrifugation is another approach that combines ultracentrifugation with sucrose density gradient^[44]^ for a successful separation of exosomes from particles and other vesicles with different densities like proteins or protein aggregates. Because sucrose is non-toxic to cells, low in viscosity and pH-neutral, the purity of exosomes obtained by sucrose is high^[45,46]^. However, this traditional method is relatively complicated to operate since the results are very sensitive to the centrifugation time. Consequently many efforts should be done to adjust this parameter properly to avoid contaminating particles in the exosomal fraction^[47]^. Cushioned Density Gradient Ultracentrifugation is a very recent modified version of this protocol leading to a maximal recovery and high purity of isolated exosomes, and preservating their structure and functionality^[48]^.

Exosomes can be also isolated by size-exclusion chromatography (SEC). This method is based in a column packed with porous beads composed by polymers. The speed of molecules passing through the beads depends on their size, thus allowing precise separation of large and small molecules^[49]^. Compared to centrifugation methods, exosomes isolated by SEC are not affected by shearing force, which can potentially change the structure of the vesicles^[50]^. Moreover, different eluting solution can be used. Nevertheless, this technique requires long running time, making difficult processing multiple samples and exosomes are eluted in a high volume, which makes necessary an additional step of concentration. Currently, SEC was reported as a suitable technique for isolation of exosomes in both serum^[51]^ and urine samples^[52]^.

Ultrafiltration membranes are another approach available to isolate exosomes from other macromolecules. Membranes with various pore sizes commercially available were designed for isolating 40-100 nm exosomes^[53]^. During the procedure, larger vesicles are removed and the exosomal population is concentrated on the filtration membrane. This technique requires a low volume sample input for obtaining high concentrated material^[54]^. The main pitfalls of this technology are: (1) the possibility of exosome adhesion to the filtration membrane with the loss of biological material; (2) the low purity; and (3) the additional force applied to pass the analyzed liquid through the membranes that can deform or cause damage to exosomes. An alternative is the tangential flow filtration, used for isolation of exosomes with well-determined size by removing free peptides and other small compounds, that showed promising results in both basic research and clinical analysis^[55]^.

In addition to use the conventional methods, some technologies previously used to extract and separate other substances were proposed for exosome isolation: polymer-based precipitation and immunoafinity. Polymer-based precipitation technique usually starts by mixing the biological fluid with polymer-containing precipitation solution [being polyethylene glycol (PEG) the most common], following by an incubation and low- speed centrifugation steps^[56]^. The precipitation with polymers results in mild effects on isolated exosomes. Polymer-based precipitation technique is a fast method with a high yield and interestingly various volumes and sources of biological fluids can be used. Despite all of these advantages, the use of polymer based precipitation solutions can result in aggregates and coprecipitation of larger nonexosomal components and together with the polymer substance present in the isolated exosomes may interfere with downstream analyses^[57]^.

Immunoafinity is based on the expression of specific protein markers expressed at the exosome membrane such as tetraspanins, integrins or adhesion molecules^[58]^. Antibodies against these markers are immobilized on coated beads (as Protein G coated Dynabeads™) which bind the exosomal specific markers (CD9, CD81, or CD63)^[59]^, afterwards the beads-exosome complexes are pelleted using magnets. The main advantage over other methods is its selectivity leading to the high purity of exosomes collected. However, this method is expensive and can only be used for small sample volumes and for high concentrated samples. The exosome yield from immunoafinity remains relatively small then reducing the potential for further analysis to low consumption methods. Nonspecific binding and potential detachment of exosomes from beads limit its interest^[60,61]^.

In the recent years, various commercial kits have been developed to separate and purify exosomes^[62,63]^. For instance, ELISA-based ExoTEST™, ExoQuick and immunoaffinitive superparamagnetic nanoparticles (ISPN) were proposed for isolating exosomes from various biological fluids^[64-65]^. ExoQuick kits do not require specific equipment, are regularly updated and the extraction efficiency is gradually improved. Interestingly, a recent study by Niu *et al.*^[66]^ compared the application of ultracentrifugation, ultrafiltration and polymer-based precipitation for exosomal isolation from human endometrial cells and showed that polymer-based method led to the lowest protein contamination^[66]^. Another study compared six kits for serum exosome purification (exoEasy, ExoQuick, Exo-spin, ME kit, ExoQuick Plus and Exo-Flow) and demonstrated that all of them can isolate exosomes but with variable efficiency and contamination by lipoproteins and albumin. Three of these methods obtained similar yield: Exo-spin, exoEasy and ExoQuick. However, exoEasy or ExoQuick Plus resulted in the highest purity^[67]^.

Besides the traditional methodologies described above, microfluidic-based exosome isolation on-a-chip systems have recently emerged. Microfluidic-based exosome isolation approaches can be divided in: (1) systems based on specific surface markers such as microfluidics-based immunoafinity capture (Mf-IAC); (2) techniques related to specific size and density such as microfluidics-based membrane filtration (Mf-F), nanowire-based traps (NTs), nano-sized deterministic lateral displacement (nano-DLD), viscoelastic flow and acoustic isolation; and (3) devices based on other physical properties, such as dielectrophoresis (DEP). Briefly, Mf-IAC consists in targeting exosomal specific surface markers either with selected antibodies immobilized in the inner surface of microfluidic chips or beads coated with antibodies^[68]^. Continual advances in nanotechnology made possible the development of several Mf-IAC devices that allow the isolation of exosomes with sensitivity and specificity while suppressing non-specific capture^[69-72]^. Mf-IAC can isolate exosomes from culture medium or body fluids. However, it is mandatory to know previously the surface antigen to target. Mf-F is based on the specific size of exosomes. The pioneer Mf-F devices were pressure- and electrophoresis-driven, which separate exosomes from undesirable particles via a nanoporous membrane with an adjustable pore size^[73]^. Further on, double-filtration approaches were developed, offering significantly higher throughput and higher purity^[74]^. The NT system is a multiscale filtration system using nanowires. NT selectively traps specific sizes of particles, and the concept is similar to size exclusion chromatography^[53]^. Nano-DLD is a pillar-array-based microfluidic device that sorts exosomes in continuous flow by taking advantage of the streamlines that particles are forced to follow due to the physical constrains imposed by the chip design or by the mechanical properties of the device^[75]^. The viscoelastic flow sorting is a continuous, size based and label-free method where exosome isolation is determined by elastic lift forces acting on particles of different sizes in a viscoelastic medium^[76]^. This method showed a very high level of purity (> 90%) and good yields. An acoustic isolation system relies on the application of differential of acoustic pressure fields that can separate small particles based on their mechanical properties including compressibility, diameter and density^[77]^. Interestingly Wu *et al*.^[78]^ recently established an acoustofluidics technique based on two modules: a cell-removal module and an EV-isolation module, that greatly simplifies the pre-processing of complex samples such as blood. Finally, DEP devices enable exosomes isolation in the presence of a non-uniform electric field. Separation is based on dielectric constant differences that determine how quickly particles move. Advantages of this approach are the few instrumentation requirements and exosome structure remains unaffected^[79]^.

Overall, microfluid-based isolating device on-a-chip provides convenience, the lab-on-a chip format can also be exploited to integrate multiple processes in a single instrument, simplifying operation and reducing the risk of cross-contamination. Moreover micro- and nano-scale devices exhibit high accuracy, precise control, lower energy consumption, cost reduction with respect to benchtop instruments and minimal sample size. A general disadvantage of all flow-induced methods is that they are based on microfluidics rules; hence, an exhaustive physical knowledge is needed for the design and operation of these devices. These microfluidic-based exosome isolation on-a-chip techniques have been reviewed in more detail in recent reviews^[68,80,81]^.

Once the exosomes have been isolated, the next step is RNA extraction. Exosomes are limited by a more rigid membrane compared to the cellular membrane due to the lipid composition, which would potentially affect the RNA extraction^[82]^. A number of conventional RNA extraction techniques are available, including phenol based techniques, combined phenol and column based approaches and pure column based methods. Eldh *et al.*[83] performed an study to determine the most suitable RNA isolation method for exosomal RNA^[83]^. They tested seven different methods: Trizol^®^, RNeasy^®^, miRNeasy, mirVana™, RNeasy^®^, modified RNeasy^®^ and miRCURY™. All tested methods were able to extract RNA with high quality but with huge variation in the yield and size distribution of exosomal RNA. miRCURY™ showed the highest total RNA yield while Trizol^®^, miRNeasy and mirVana™ showed the lowest total RNA yield in exosomes. Moreover, the relative presence of smaller and larger RNA molecules was dependent on the used method. In short, column based RNA extraction techniques could extract RNA with a broad size distribution, whereas the pure phenol and the combined phenol-column extraction techniques seemed to be more efficient at extracting small RNA rather than total RNA. In terms of RNA purity Trizol^®^, modified RNeasy^®^, miRNeasy and mirVana™ showed a reduced purity comparing to the other strategies, probably due to residual phenol/guanidine. Since the different isolation methods give such an extensive variation in exosomal RNA yield and pattern, it is of main importance to select the RNA extraction method according to the aims of the research, especially in exosome research, since there is a relatively limited amount of exosomes from different body fluids.

Finally, for the detection of lncRNA, after exosomal RNA isolation, several methods are in continuously innovation and improvement [Table 2]. Briefly, northern blot was initially the main methodology used for detection and quantification of RNA in cancer cells and tissues^[84]^. With the rapid development of next-generation sequencing (NGS) technology, the high-throughput sequencing has been widely used for discovery of candidate genes and lncRNAs^[85]^. Similarly, lncRNA microarray is used to analyze lncRNAs differentially expressed^[86]^. However, the cost of NGS and microarray analysis is relatively high, and the high amounts of data generated do not allow their use in clinical practice. Thus, nowadays the quantitative reverse transcription polymerase chain reaction (RT-qPCR) represents the most common detection method for detecting lncRNA with a relatively simple procedure^[87]^.

**Table 2:** Methods used for detecting lncRNAs

Method	Principle	Advantages	Disavantage
Northern Blot	- Electrophoresis and detection with specific probe	- Fast, low-tech, cheap - Alternative splicing products can be detected - Both quantitative and qualitative method - High specific	- High risk of sample degradation - Low sensitivity - Only known sequences detected
RT-qPCR	- Transcript amplification and fluorescence signal detection after specific probe hybridization	- Cost-effective - Time-efficient - High sensitivity and specificity, l - Low amount of starting material - Results easy and fast to obtain	- Splicing products no detected - Nonspecific binding - Maximum 4 different mRNAs can be detected simultaneously - Only known sequences detected
Microarrays	- Molecular hybridization to detect the expression levels	Multiple mRNAs can be analyzed in the same experiment, well defined and standardized protocols, relatively low cost	- Detection of known sequences - Non-specific hybridization - No identification of mRNA variants - High variability of low expressed mRNAs
RNA-seq	- Next generation sequence based	- Independency from previous sequence information - High dynamic range - Several isoforms of mRNA can be detected - Low amount of starting material is required	- High cost - Complex analysis of data

In the following paragraphs, we will review the principles of the different methodologies for lncRNA detection as well as their pros and cons. Northern blot was more used in the past, however there are still some reports using this technique for lncRNA detection^[84,88]^. Northern blotting involves the use of electrophoresis to separate RNA samples by size, and detection with a hybridization probe complementary to part of or the entire target sequence^[89]^. Some advantages of using northern blotting include: relatively fast, low -tech and cheap procedure; ability to detect the size of RNA fragment; observation of alternative splicing products; measurement of both RNA quality and quantity. Northern blot is a high specific method that reduces false positives and gives the opportunity to store membranes that can be reprobed years after blotting. Northern blot also has some pitfalls like: high risk of sample degradation by RNases; its low sensitivity and the use of some chemicals, such as formaldehyde, radioactive material or ethidium bromide used which can have harmful effects on health^[90]^.

The most used methodology for lncRNA is RT-qPCR^[34,36,91]^, which allows the quantification of lncRNAs expression in the sample thanks to transcript amplification and further fluorescent signal detection^[92]^. It is a cost-effective and time efficient method, moreover it shows a high sensitivity and specificity, low amount of starting RNA is required, and the results are fast and easy to obtain, since it is not required any post PCR processing or data processing. However RT-qPCR has also some limitations such as: the amplicon size cannot be detected; maximum of four lncRNAs can be detected simultaneously; and non-specific binding can occur if SYBR^®^ green is used, among others^[93]^. Another technique commonly used to identify lncRNAs are microarrays^[94,95]^, which consist of a library of DNA specific sequences (known as probes) immobilized in a grid. The isolated mRNA is reversed transcribed into cDNA incorporating fluorescent probes and hybridized to the microarray. Only the cDNAs complimentary to the probes, and thus the ones that are expressed, are detected by the laser scanner^[96]^. This technology provides data for multiple lncRNAs in the same experiment, the protocols are well defined and standardized, and it is a relatively low-cost method. But it has also some disadvantages like: the analysis is only possible for known lncRNAs; the hybridization can be non-specific, thus giving false positives; high variability for low expressed genes; and not identification of lncRNAs sequence variants^[97]^. RNA-seq is probably the most accurate technique to reveal the presence and quantify lncRNA in a biological sample at a given time^[85]^. The fact that RNA-seq is not dependent on previous sequence information is a major strength, it has a high dynamic range and several isoforms of lncRNAs can be detected. Disadvantages are more related to the high cost of the technology and complex analysis process that requires high expertise of researchers^[94]^. Interestingly Lane *et al*.^[57]^ recently published a book with extended information about methodologies for exosomal isolation and identification^[57]^.

## Clinical value of using exosomal lncRNAs as liquid biopsies

The continuous development of diagnostic techniques provides a technical basis for liquid biopsy in patients with cancer and for quantitative detection of circulating exosomal lncRNAs. By comparing the expression of lncRNA between normal population and cancer patients, the exosomal lncRNA-based liquid biopsy may be used as a diagnostic technique^[98]^. Several studies validated the utility of exosomal lncRNAs as minimally invasive diagnostic and prognostic marker in all kind of body fluids [Table 3]. Although there is no systematic study on the difference of circulating lncRNAs between serum-derived and plasma-derived exosomes, the method of blood sample testing is widely used in the clinic because of the accessibility and relative high stability of the biological material and the minimally invasive operation requested. In this paragraph, we will review some of the most studies recently published [Table 3].

**Table 3:** Clinical investigations using exosomal lncRNAs as prognostic and diagnostic biomarkers

Type of cancer	Body fluid	Main findings	Ref.
Colorectal cancer (CRC)	Serum	Correlation of exoCRNDE-h levels with CRC regional lymph node metastasis and distant metastasis. ExoCRNDE-h levels can differentiate CRC from patients with benign disease and healthy donors. *n* = 148	[99]
CRC	Plasma	Upregulation of exoLNCV6_116109, 98390, 38772, 108266, 84003, and 98602 in early CRC stages. *n* = 100	[100]
Non-small cell lung cancer (NSCLC)	Serum	Lower expression levels of Exo-GAS5 in NSCLC patients than healthy donors. Higher Exo-GAS5 in early stages. Exo-GAS5 is a better prognostic marker than carcinoembryonic antigen (CEA). Lower expression of Exo-GAS5 correlates with larger tumors. *n* = 104	[36]
Glioblastoma multiforme (GBM)	Serum	Exo HOTAIR expression higher in GBM patients. *n* = 83	[33]
Breast cancer (BC)	Serum	Exo HOTAIR higher expressed in BC patients than in healthy donors. High expression of Exo HOTAIR correlates with poor disease free survival (DFS), overall survival (OS) and poor response to neoadjuvant chemotherapy (CT) and tamoxifen hormone therapy. Exo HOTAIR better prognostic and diagnostic biomarker than CA 15-3. *n* = 30	[101]
Bladder cancer	Urine	Exo MALAT1, PCAT-1 and SPRY4-IT1 overexpressed in bladder cancer patients. High expression of PCAT-1 and SPRY4-IT1 correlates with TNM stage. *n* = 160	[35]
Bladder cancer	Urine	Exo UCA1-201 expression levels can discriminate between patients suffering from bladder cancer, nonmalignant urinary related disorders and healthy donors. *n* = 108	[102]
Oral Squamous Cell Carcinoma (OSCC)	Saliva	OSCC patients expressed MALAT-1. Higher HOTAIR expression in patients with lymph node metastasis. *n* = 20	[34]
NSCLC	Serum	Exo RP11-838N2.4 as a potential target to predict response of NSCL patients to erlotinib treatment. *n* = 78	[103]
BC	Serum	Exo SNHG14 higher expressed in resistant patients to trastuzumab treatment. Increased Ex SNHG14 expression associated with metastasis and cardiac toxicity. *n* = 72	[104]
Renal Cell Carcinoma (RCC)	Plasma	High levels of Exo lnARSR associated to sunitinib resistance. LnARSR have to be packed into exosomes to exert resistance. Exo lnARSR is a therapeutic target. *n* = 74	[17]

Liu *et al*.^[99]^ analyzed the utility of the exosomal colorectal neoplasia differentially expressed-h (CRNDE-h) non-coding RNA in a cohort of 148 patients suffering from a variety of colorectal cancer (CRC) compared to control volunteers. They isolated RNA from serum-purified exosomes and measured the CRNDE-h expression levels by RT-qPCR. Their main findings indicated a significant correlation of the exosomal CRNDE-h lncRNA levels with the CRC regional lymph node/distant metastatic status. Moreover, ROC analysis showed the possibility to distinguish CRC patients, colorectal benign diseases and healthy individuals with 70.3% sensitivity and 94.4% specificity, which was superior to the traditional CEA tumor marker. In conclusion, detection of exosomal CRNDE-h lncRNA has a high potential as a noninvasive serum-based tumor marker for diagnosis and prognosis of CRC, for developing new therapeutic strategies^[99]^. The survival of CRC patients is closely related to the stage at diagnosis; therefore, there is an urgent need for early diagnosis for CRC prevention and successful treatment. The study performed by Hu *et al*.^[100]^ aimed to identify potential exosomal lncRNAs that may use as early stage biomarkers for CRC. Microarray studies identified a subset of 6 lncRNAs (LNCV6_116109, LNCV6_98390, LNCV6_38772, LNCV_108266, LNCV6_84003, and LNCV6_98602) significantly higher expressed in the plasma of CRC patients than in negative control individuals. They isolated exosomal RNA from the plasma of 50 CRC patients and 50 healthy donors for validation of these potential biomarkers by RT-qPCR. The authors found a correlation between the high levels of expression of these markers and the presence of CRC. Furthermore, they analyzed the expression patterns in each stage of CRC and found the 6 lncRNAs significantly upregulated in stage I/II of CRC disease. These authors concluded that the 6 lncRNA panel can be used as biomarkers for non-invasive screening of CRC with high sensitivity and specificity^[100]^. Li *et al*.^[36]^ also carried out an study to determine the usefulness of the exosomal GAS5 lncRNA (Exo-GAS5) as a biomarker for early stage non-small cell lung cancer (NSCLC) diagnosis. They analyzed by RT-qPCR the expression of Exo-GAS5 isolated in the serum from 64 NSCLC patients and 40 healthy donors, and found a lower expression in the former group. Moreover, those patients in early stage presented higher Exo-GAS5 levels compared with advanced-stage patients. The authors also demonstrated a better performance of the Exo-GAS5 than the conventional tumor marker CEA to distinguish NSCLC patients at early stages from healthy volunteers. Finally, they identified a significant association between lower Exo-GAS5 expression with larger tumor size and advanced TNM stage^[36]^.

Glioblastoma multiforme (GBM) is the most common and aggressive malignant adult primary brain tumor. After tumor resection, patients are monitored by magnetic resonance imaging (MRI)^[91]^. However, this imaging technique is unable to distinguish between true progression from pseudo-tumor progression, which may have consequences in clinical decisions. Tan *et al*.^[33]^ designed a study including 43 GBM patients and 40 healthy donors to determine the feasibility of using exosomal HOTAIR lncRNA as a serum biomarker in conjunction with MRI to distinguish pseudo-progression and true progression. They assessed the HOTAIR expression levels by RT-qPCR and observed an increase in HOTAIR both in whole serum and purified exosomes but not in serum supernatant depleted of exosomes of GBM patients in contrast to control group. Moreover, they confirmed this finding by single molecular sequencing (SMS) analysis. Therefore, they proposed the detection of exosomal HOTAIR lncRNA in the serum of GBM patients as a noninvasive biomarker to detect GBM growth or recurrence^[33]^. Similarly Tang *et al*.^[101]^ also showed the correlation of high serum exosomal HOTAIR levels with poor survival and poor response to chemotherapy in breast cancer patients. Briefly, they compared serum exosomal HOTAIR expression levels between 15 breast cancer patients and 15 healthy individuals, demonstrating a significantly higher expression in breast cancer patients than in healthy controls. They observed a marked decrease of serum exosomal HOTAIR lncRNA levels after 3 months of surgery and ROC analyses showed that serum exosomal HOTAIR had a significant greater capacity than the CA 15-3 antigen as a diagnostic and prognostic biomarker. In addition, results for the survival analysis revealed that high expression of exosomal HOTAIR lncRNA led to a worse disease-free survival and overall survival than low expression. Finally, the analysis of exosomal HOTAIR lncRNA expression in patients before and after treatment suggested that high levels may lead to a poor response to neoadjuvant chemotherapy and to tamoxifen hormone therapy.

Exosomal lncRNAs can be also isolated from other body fluids rather than the most common, such as plasma or serum. For instance, Zhan *et al*.^[35]^ validated a panel of three lncRNAs (MALAT1, PCAT-1 and SPRY4-IT1) by RT-qPCR, in urine exosomes (UE) of 80 bladder cancer patients and 80 healthy controls, and found a significant overexpression in bladder cancer patients. The verification of the diagnostic capacity indicated a much better performance for this panel than for the urine cytology, which is widely used in clinical practice. Moreover, they identified a significant correlation between the overexpression of UE-derived PCAT-1 and SPRY4-IT1 and the advanced TNM stage^[35]^. The utility of UE as a liquid biopsy in bladder cancer was confirmed by Yazarlou *et al*.^[102]^. The study included a total of 59 BC patients, 24 healthy individuals and 25 patients with non-malignant urinary related disorders. They evaluated expression of several lncRNAs in urinary isolated exosomes by RT-qPCR in all the study participants. They found a significantly higher expression of LINC00355, UCA1-203, and MALAT1 and a decreased UCA1-201 expression in bladder cancer patients compared to the other two groups. Among all these lncRNAs, UCA1-201 transcript levels had the best performance to discriminate between bladder cancer from normal/nonmalignant disease samples, and thus indicating its potential use as a prognostic biomarker in BC^[102]^.

Saliva is another source of lncRNA and liquid biopsy that has been proposed for clinical application. Tang *et al*.^[34]^ detected by RT-qPCR the presence of the MALAT-1 lncRNA in the saliva of all oral squamous carcinoma patients analyzed (*n* = 20), despite mRNA expression was not significantly different between metastatic and non-metastatic samples. However, HOTAIR was detected mostly in patients with lymph node metastasis, compared with those without metastasis. These results indicated a promising use lncRNAs in saliva as a noninvasive and rapid diagnostic tool for the diagnosis of oral cancer, but a higher number of samples must be included to confirm that results^[34]^.

In addition to their value as diagnosis and prognosis biomarkers in cancer, exosomal lncRNAs were involved in drug resistance of tumor cells, suggesting a clinical application in cancer-targeted therapy. A recent study demonstrated the therapeutic benefit of erlotinib in patients suffering from NSCLC in large randomized phase III studies, however the majority of these patients exhibited erlotinib-refractory disease, acquiring chemoresistance. In this context, there is an urgent need to elucidate the mechanisms of erlotinib resistance and to discover reliable biological targets that play important role in erlotinib resistance^[105]^. Zhang *et al*.^[103]^ analyzed the expression level of exosomal RP11-838N2.4 lncRNA by RT-qPCR in 78 serum samples from patients with advanced NSCLC receiving erlotinib treatment^[103]^. They identified the involvement of the exosomal RP11-838N2.4 lncRNA in the modulation of chemotherapeutic responses, suggesting its potential role as a novel target for prediction of NSCLC treatment with erlotinib.

Trastuzumab is effective used for early stage treatment of breast cancer patients bearing positive HER2 mutations, however, after a period of exposure, some patients acquire resistance. Then, there is a clear need for useful therapeutic biomarkers able to predict chemoresponses to treatment with trastuzumab^[106]^. Several *in vitro* assays performed by Dong *et al*.^[104]^ confirmed that the exosomal SNHG14 lncRNA was essential for trastuzumab resistance in breast cancer. They validated this statement in serum samples from a cohort of 72 patients with advanced HER2+ breast cancer treated with trastuzumab. Their results showed an increased serum exosomal SNHG14 lncRNA expression level, determined by RT-qPCR, in patients who did not respond to the treatment compared to good responders. ROC analysis demonstrated a high sensitivity and specificity of SNHG14 lncRNA as a potential diagnostic biomarker for trastuzumab breast cancer treatment. Moreover, they also observed an increased expression of exosomal SNHG14 lncRNA was markedly associated with distant metastasis, lymph node metastasis and cardiac toxicity^[104]^.

In renal cell carcinoma (RCC) nearly 30% of patients develop recurrence and metastasis after tumor resection^[107]^. Sunitinib, which has potent anti-angiogenic effects and direct antitumor activities, was proposed as an effective therapy for RCC patients. However, the development of sunitinib resistance was observed resulting in failure of sunitinib and reduced survival rate^[108]^. The *in vitro* studies carried out by Qu *et al.*[17] pointed out that high level of lncARSR gene were responsible for sunitinib resistance. They confirmed these findings by comparing the expression of this lncRNA between responding and non-responding patients, both in tissues and plasma (*n* = 74). Additional analysis let to conclude that high lncARSR levels in plasma correlated with poor sunitinib response in RCC patients. They investigated further into the mechanism of resistance to sunitinib indicating that it was required lncARSR to be packed in exosomes to exert resistance. Moreover, they observed that lncARSR incorporated into exosomes and transmitted to sensitive cells, resulted in sunitinib resistant cells, thereby disseminating drug resistance^[17]^. All together the authors demonstrated not only potential utility of exosomal lncARSR as a clinical biomarker to sunitinib response but also as a therapeutic target to overcome sunitinib resistance and improve the clinical benefits in RCC patients. Thus, liquid biopsy by detecting exosomal lncRNA provides a minimally invasive approach, a real-time monitoring of drug response, and accurate information for clinicians to administer a reasonable medication.

## Discussion and future perspectives

LncRNAs were confirmed to be closely related to the development of cancer^[6]^, and exosomal lncRNAs can be stably detected in human body fluids because of the protection of exosomes^[29]^. This together with the continuous development of lncRNA detection techniques has promoted exosomal lncRNA-derived liquid biopsy to become a novel approach in the diagnosis, prognosis, and treatment of cancers^[109]^. As described in the section 4 of this review the studies on exosomal lncRNAs are accumulating, however its clinical use is not really extended yet. Indeed, liquid biopsies are not currently considered as a standard operating procedure for the diagnosis of cancers and for the characterization of the disease. Instead it is used as a complimentary test to tissue biopsy^[110]^. However, cancer patients are often unable to undergo highly invasive examinations because of their poor physical condition, thus a minimally invasive, reproducible, real-time detection is extremely desirable. Despite the numerous studies in liquid biopsy, clinical validations are mandatory to assess its accuracy explaining why its use is not very widespread in medical practices.

To use exosomal lncRNAs as novel biomarkers, high purity and accuracy techniques for their isolation and detection are required. Currently, many methods were developed for and each of them has many advantages and disadvantages which must be evaluated. The method used should be selected according the objectives of the study.

Many challenges regarding exosomal lncRNAs need to be confronted. For instance, physiological and pathological roles of exosomes and exosomal lncRNAs in the tumor microenvironment remain to be further explored. In addition, the heterogeneity of exosomes in body fluids may be a drawback to their use as biomarkers, since can lead to false negatives or positives in cancer diagnosis. Prior to the application in the clinical context, standardized methods of preparation of exosomal lncRNAs from biofluids are mandatory. Indeed, many pre-analytical factors such as time of fluid collection, fluid preservation or RNA isolation protocol among others, influence RNA abundance levels in body fluids both inside and outside extracellular vesicles^[111]^, affecting RNA liquid biopsy performance. Thus, the exact contribution of these variables must be rigorously assessed. A recent initiative for this purpose is “The extracellular RNA quality control (exrnaqc) study” carried out by Decock *et al*.^[112]^. The most appropriate source (nature of the biological fluids) of exosomal lncRNAs must be defined^[29]^. For instance, results from lncRNAs extracted from whole blood should be interpreted cautiously due to lncRNAs contained in blood cells and/or circulating cancer cells and/or systemic inflammatory context that could result in important deviations^[113]^. The individual variability/heterogeneity in lncRNA tumor expression patterns (intra- and inter-tumor heterogeneity^[114]^) should be considered as well as other external variables such as diet, physical activity or drugs^[115,116]^. Technological improvements of lncRNA measurement are then needed to improve its sensitivity, normalization as well as data analysis. For instance, total RNA sequencing methods are still difficult to implement in many research laboratories and would benefit from further technological improvement. Notably, in the vast majority of the studies that we found in the literature, the detection of lncRNA is currently carried out by RT-qPCR, however the cost related to the yield is still too high. A similar emerging technology is digital PCR (dPCR) characterized by a highest robustness and technical reproducibility as well as an improved sensitivity (30-fold) and accuracy (10-fold)^[117]^. Therefore, dPCR represents a very promising method for detecting lncRNAs in liquid biopsies with low nucleic acid content^[118]^. Recent studies already reported the use of dPCR to detect exosomal lncRNAs^[119,120]^. However, data normalization is required to reduce the variation associated with sample collection, processing and measurement. Despite of some studies on exosomal lncRNAs showed a better performance than the conventional biomarkers in terms of prognosis and diagnosis^[36,101]^, further investigations are necessary to confirm if lncRNAs can be used as independent biomarkers or should be used in combination with the existing ones. Multicentric clinical studies will be also required before to use lncRNAs as diagnostic biomarkers.

## Conclusion

The recent discovery of the association of exosomal lncRNAs and cancer progression has attracted the attention of researchers and opened up exciting prospects for diagnostics, prognostics and drug response monitoring. Elucidating the exact roles of exosomal lncRNAs in tumorigenesis, metastasis, and drug resistance, as well as the development and application of standard operating procedures, are areas of considerable interest to offer a more sensitive and accurate biomarker for early cancer detection or to improve the efficiency of existing clinical biomarkers.

## Declarations

### Copyright

© The Author(s) 2019.
